# p38γ Mitogen-Activated Protein Kinase Is a Key Regulator in Skeletal Muscle Metabolic Adaptation in Mice

**DOI:** 10.1371/journal.pone.0007934

**Published:** 2009-11-20

**Authors:** Andrew R. Pogozelski, Tuoyu Geng, Ping Li, Xinhe Yin, Vitor A. Lira, Mei Zhang, Jen-Tsan Chi, Zhen Yan

**Affiliations:** 1 Department of Medicine, Duke University Medical Center, Durham, North Carolina, United States of America; 2 Institute for Genome Sciences and Policy, Duke University Medical Center, Durham, North Carolina, United States of America; 3 Department of Molecular Genetics and Microbiology, Duke University Medical Center, Durham, North Carolina, United States of America; 4 Department of Medicine-Cardiovascular Medicine, University of Virginia, Charlottesville, Virginia, United States of America; 5 Center for Skeletal Muscle Research at Robert M. Berne Cardiovascular Research Center, University of Virginia, Charlottesville, Virginia, United States of America; McMaster University, Canada

## Abstract

Regular endurance exercise induces skeletal muscle contractile and metabolic adaptations, conferring salutary health benefits, such as protection against the metabolic syndrome. The plasticity of skeletal muscle has been extensively investigated, but how the adaptive processes are precisely controlled is largely unknown. Using muscle-specific gene deletion in mice, we now show that p38γ mitogen-activated protein kinase (MAPK), but not p38α and p38β, is required for endurance exercise-induced mitochondrial biogenesis and angiogenesis, whereas none of the p38 isoforms are required for IIb-to-IIa fiber-type transformation. These phenotypic findings were further supported by microarray and real-time PCR analyses revealing contractile activity-dependent p38γ target genes, including peroxisome proliferator-activated receptor γ co-activator-1α (*Pgc-1*α) and vascular endothelial growth factor (*Vegf*), in skeletal muscle following motor nerve stimulation. Gene transfer-mediated overexpression of a dominant negative form of p38γ, but not that of p38α or p38β, blocked motor nerve stimulation-induced *Pgc-1α* transcription. These findings provide direct evidence for an obligated role of p38γ MAPK-PGC-1α regulatory axis in endurance exercise-induced metabolic adaptation, but not contractile adaptation, in skeletal muscle.

## Introduction

Endurance exercise induces profound adaptive changes in skeletal muscles, including but not limited to IIb-to-IIa fiber-type transformation, and mitochondrial biogenesis (increased mitochondrial size and density) and angiogenesis (increased capillary density), conferring the functional characteristics of a more oxidative phenotype with greater endurance capacity. A general role of the p38 mitogen-activated protein kinase (MAPK) in exercise-induced skeletal muscle adaptation has been implicated in studies where muscle contractile activity activates the p38 MAPK pathway [1], [2], [3], [4], [5] as well as the peroxisome proliferator-activated receptor γ co-activator-1α (PGC-1α) activity [6] and gene expression [1], [7], [8], [9], [10]. Specifically relevant to endurance exercise-induced muscle adaptation, it has been shown that transgenic mice with forced exogenous expression of constitutively active p38 MAPK activator, MAPK kinase 6 (MKK6), have enhanced mitochondrial biogenesis [8], and functional interactions of the p38 MAPK/ATF2 and PKD/HDAC5/MEF2 signaling modules with the *Pgc-1a* promoter confer the transcriptional control [11], [12]. Therefore, the p38 MAPK-PGC-1α regulatory axis presents a possible mechanism for endurance exercise-induced skeletal muscle adaptation with two important questions related to endurance exercise training remained to be addressed: Which of the three isoform(s) of p38 MAPK (α,β,γ) in skeletal muscle is functionally involved in skeletal muscle adaption? Which of the skeletal muscle adaptive processes (mitochondrial biogenesis, angiogenesis and fiber-type transformation) is controlled by the endogenous components of the p38 MAPK-PGC-1α regulatory axis?

In this study, we employed two independent loss-of-function molecular genetic approaches in physiological models of endurance exercise in mice to delineate the role of the p38 MAPK in skeletal muscle adaptation. Our findings confirmed for the first time at both biochemical and transcriptional levels that p38γ is required for endurance exercise-induced mitochondrial biogenesis and angiogenesis, but not for IIb-to-IIa fiber-type transformation.

## Results

### Muscle-Specific Deletion of the p38 Genes Does Not Affect Endurance Exercise-Induced Contractile Adaptation

**Figure 1:**
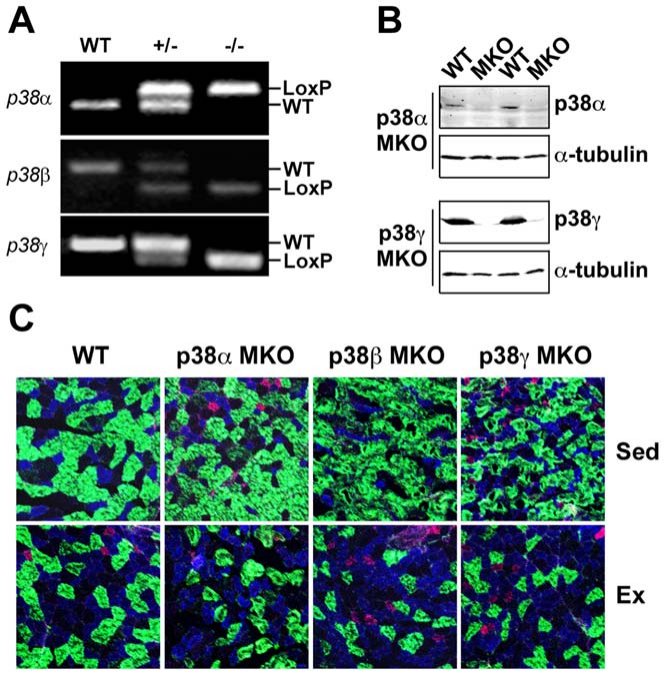
Muscle-specific deletion of the p38α, p38β or p38γ gene does not affect endurance exercise-induced fiber-type transformation. Wild type, p38α, p38β, and p38γ MKO mice were subjected to 4 weeks of voluntary running (Ex) or sedentary cage activity (Sed) followed by fiber-type and immunoblot analyses in plantaris muscles. A) PCR of genomic DNA with the appropriate primers (see Materials and Methods) for the loxP flanked p38 alleles in wild type (WT), heterozygous (+/−) and homozygous (−/−) mice with loxP-flanked p38 alleles. Only the homozygous mice with the *Cre* transgene (not shown) were considered p38 muscle-specific knockout (MKO) mice; B) Immunoblot analysis for p38α and p38γ protein in plantaris muscles of p38 MKO mice in comparison with the wild type littermates. α-tubulin was used as a loading control; and C) Images of immunofluorescence staining of plantaris muscle sections with antibodies against myosin heavy chain IIb (Green), IIa (Blue) and I (Red). Appreciable increases in the percentage of type IIa fibers with concurrent decreases in type IIb fibers are noted in Ex group compared with Sed group among all four genetic backgrounds. The quantitative data is presented in Table 1.

To ascertain the functional importance of the p38 isoforms in endurance exercise-induced skeletal muscle adaptation, we crossbred myogenin*-Cre* transgenic mice (generously provided by E. Olson) [13] with mice in which the p38 alleles were flanked by *loxP* sites (generously provided by Boehringer Ingelheim Pharmaceuticals, Inc.). Myogenin is a basic helix-loop-helix (bHLH) transcription factor that is required for skeletal muscle differentiation [14], and therefore expression of the *Cre* gene under the control of the myogenin promoter allows for deletion of a gene of interest specifically in skeletal muscle [13]. Following two rounds of crossbreeding, we obtained muscle-specific p38 knockout mice (MKO) (Fig. 1A). The efficacy of the *Cre/loxP* genetic deletion system was confirmed at the protein livels by immunoblot analysis, showing that p38α and p38γ protein expression was completely ablated in p38α MKO and p38γ MKO mice, respectively (Fig. 1B). The specific antibodies for p38β did work in our hands; however, we expect similar degrees of ablation of p38β for p38β MKO mice as the exactly same genetic approach was employed. When we subjected wild type, p38α MKO, p38β MKO and p38γ MKO mice (male, 8 weeks of age) to voluntary running (4 weeks), the daily running distance for each mouse line increased gradually and reached a steady-state level by the end of the second week. The average daily running distance during the experimental period was not statistically different among different mouse groups (p = 0.57) (Fig. S1A), and the trend of increases in the heart weight induced by voluntary running was similar among different groups, consistent with a similar training effect (Fig. S1B).

**Table 1:** Fiber type transformation in plantaris muscles in response to voluntary running in wild type and p38 MKO mice.

	WT		*p38α* MKO		*p38β* MKO		*p38γ* MKO	
	Sed	Ex	Sed	Ex	Sed	Ex	Sed	Ex
	(n = 6)	(n = 8)	(n = 4)	(n = 5)	(n = 4)	(n = 5)	(n = 7)	(n = 5)
I (%)	1.9±1.0	1.6±0.5	0.7±0.4	0.4±0.2	0.1±0.1	1.9±0.7	2.0±0.7	1.6±0.5
IIa (%)	23.2±1.5	32.4±2.2*	25.4±4.6	37.8±4.1*	14.4±1.9	36.6±2.4***	26.8±3.1	37.4±2.9*
IId/x (%)	31.3±0.8	28.6±1.6	31.6±2.2	29.8±2.7	31.8±0.9	31.1±0.9	28.5±1.5	30.4±1.1
IIb (%)	43.6±2.4	37.5±2.2	42.3±6.6	32.1±2.6*	53.7±2.6	30.5±1.9***	42.7±3.4	30.7±3.3**

Values are means±SE in wild type (WT) and p38 MKO mice under sedentary (Sed) and exercise (Ex) conditions. *, ** and *** denote P<0.05, 0.01 and 0.001, respectively, vs. sedentary group of the same genotype.

To determine if the p38 MAPK pathway is required for endurance exercise-induced fiber-type transformation, we performed fiber-type analysis. None of the deletions of the p38 genes had a significant impact on endurance exercise-induced fiber-type transformation in plantaris muscles as shown by increases in the percentage of type IIa myofibers (Fig. 1C and Table 1) despite the fact that p38β MKO had a lower percentage of type IIa fibers than the wild type mice before endurance exercise training. The immunofluorescence finding was further confirmed by immunoblot analysis, showing significant increases of myosin heavy chain IIa protein expression for each of the mouse lines following 4 weeks of exercise (Fig. S2). Altogether, these findings provide direct evidence that none of the p38 isoforms in skeletal muscle is required for endurance exercise-induced IIb-to-IIa fiber-type transformation.

### Muscle-Specific Deletion of the p38γ Gene, but Not That of the p38α and p38β Gene, Attenuates Endurance Exercise-Induced Metabolic Adaptation

**Figure 2:**
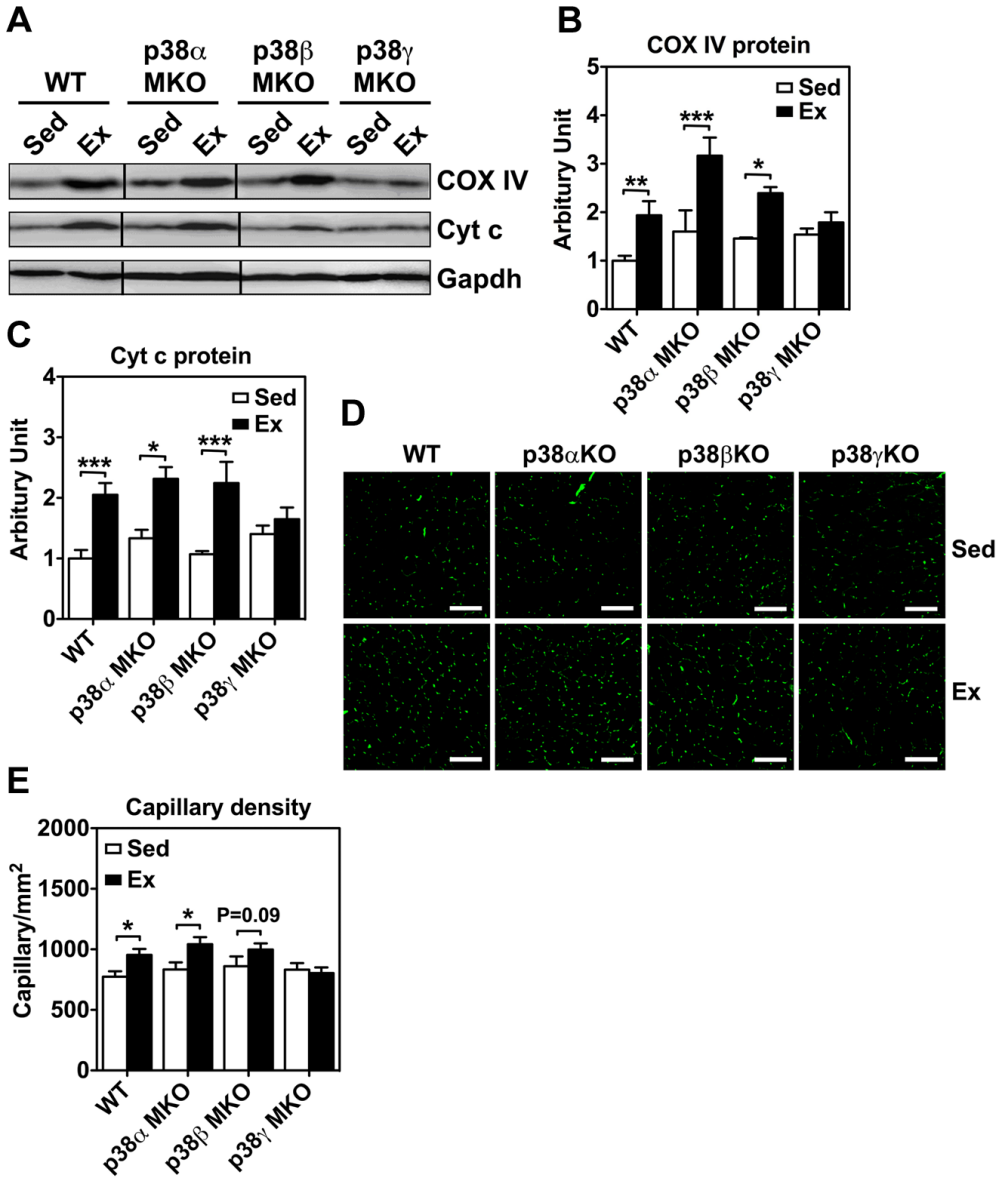
Muscle-specific deletion of the p38γ gene attenuates endurance exercise-induced metabolic adaptation. Wild type, p38α, p38β, and p38γ MKO mice were subjected to 4 weeks of voluntary running (Ex) with sedentary mice (Sed) as control, and plantaris muscles were harvested for immunofluorescence-based capillary density and immunoblot analyses. A) Representative images of COX IV and Cyt c proteins in plantaris muscle. Lines divide the images from different gels. Appreciable increases in COX IV and Cyt c are noted in Ex group compared with Sed group in WT, p38α MKO and p38β MKO mice, but not in p38γ MKO mice; B) Quantitative analysis of COX IV in plantaris muscles (n = 5–8); C) Quantitative analysis of Cyt c in plantaris muscles (n = 5–8). * and *** denote p<0.05 and 0.001, respectively. D) Images of immunofluorescence staining of plantaris muscle sections with antibodies against CD31. Appreciable increases in capillary density are noted in Ex group compared with Sed group in WT, p38α MKO and p38β MKO mice, but not in p38γ MKO mice; and E) Quantitative analysis of capillary density in the whole plantaris muscles (n  =  5–8). * denotes p<0.05.

To elucidate the functional role of p38 MAPK in endurance exercise-induced mitochondrial biogenesis, we performed immunoblot analysis for cytochrome c oxidase IV (COX IV) and cytochrome c (Cyt c) protein expression. These proteins are important components of the electron transport chain, and their expression levels are indicative of mitochondrial biogenesis [15], [16], [17]. Wild type, p38α MKO and p38β MKO mice showed 1.9- (p<0.01), 2.0- (p<0.01) and 1.7-fold (p<0.05) increases, respectively, in COX IV protein expression in plantaris muscle after endurance exercise training, but p38γ KO mice failed to show a significant change (1.2-fold; p = 0.31) (Fig. 2A and 2B). Similarly, endurance exercise training induced a 2.0- (p<0.01), 1.7- (p<0.05) and 2.1-fold (p<0.01) increases in Cyt c protein expression in wild type, p38α MKO and p38β MKO mice, respectively, but not in p38γ MKO mice (1.1-fold; p = 0.46) (Fig. 2A and 2C). These findings suggest that p38γ, but not p38α and p38β, is required for endurance exercise-induced mitochondrial biogenesis in skeletal muscle.

**Figure 3:**
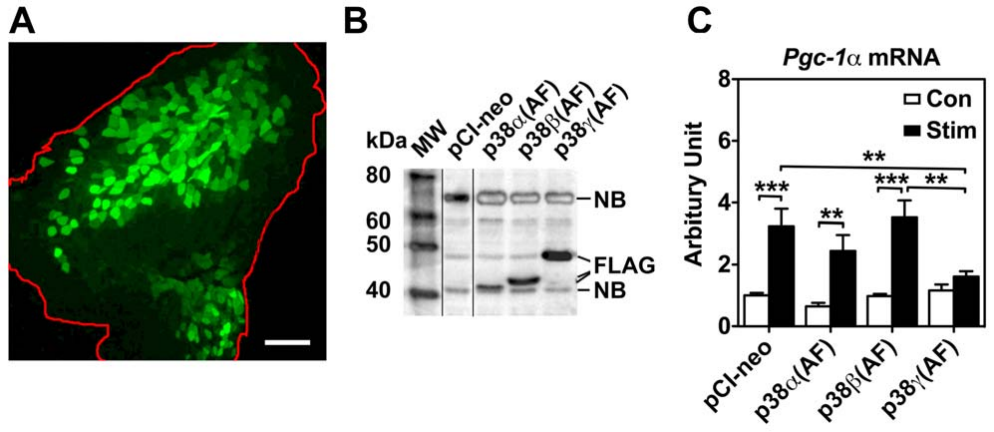
Overexpression of dominant negative p38γ, but not that of p38α and p38β, blocks contractility-induced Pgc-1α mRNA expression. Plasmid DNA (100 µg) containing an empty vector (pCI-neo) or FLAG-tagged dominant negative forms of p38α (p38α (AF)), p38β (p38β(AF)), or p38γ (p38γ(AF)) were injected into right and left tibialis anterior muscles followed by electric pulse-mediated gene transfer (described in Materials and Methods). After 10 days of recovery, motor nerve stimulation (10 Hz, 2 hours) was performed for the left tibialis anterior muscle, and both the stimulated and the contralateral control tibialis anterior muscles were harvested for analyses for transgene expression, signaling molecule activation and *Pgc-1α* mRNA expression. A) Fluorescence image showing the efficacy (∼60%) of gene transfer in tibialis anterior muscle (outline in red). The scale bar equals 200 µ; B) Immunoblot analysis for the tibialis anterior muscles 10 days after gene transfer showing expression of FLAG-tagged p38α (AF), p38β(AF), and p38γ(AF) compared with the tibialis anterior muscle transfected with pCI-neo control plasmid. Lines divide images from different gels. Non-specific bands (NB) were labeled; and C) Real-time PCR analysis showing that motor nerve stimulation results in significant induction of *Pgc-1α mRNA* in tibialis anterior muscles transfected with pCI-neo, p38α (AF) or p38β(AF), which is blocked by overexpression of p38γ(AF). The data was normalized by *18S* ribosomal RNA (n = 6–12). *, ** and *** denote p<0.05, 0.01 and 0.001, respectively.

Endurance exercise training also induces angiogenesis in skeletal muscle as a metabolic adaptation, improving blood flow capacity among myofibers that are recruited during exercise [18], [19]. More recently, it has been shown that hypoxia-induced angiogenesis is under the control of PGC-1α through regulation of the vascular endothelial growth factor (Vegf) gene expression [20]. To determine if p38 MPAK is involved in endurance exercise-induced angiogenesis, we performed immunofluorescence analysis by using specific antibodies against platelet endothelial cell adhesion molecule-1 (PCAM-1, CD31) to evaluate capillary density in skeletal muscle following voluntary running [19]. Voluntary running induced moderate or a trend of increases of capillary density in plantaris muscles in wild type (p<0.05), p38α MKO (p<0.05) and p38β MKO mice (p = 0.09), but not in p38γ MKO mice (p = 0.72) (Fig. 2D and 2E). We later confirmed attenuated expression of *Vegf* mRNA in response to motor nerve stimulation in skeletal muscle (Fig. 3A). Thus, muscle-specific deletion of the p38γ gene, but not that of the p38α and p38β genes, attenuates endurance exercise-induced angiogenesis in skeletal muscle.

### Dominant Negative p38γ, but Not p38α or p38β, Blocks Endurance Exercise-Induced Pgc-1α mRNA Expression in Skeletal Muscle

To further determine if p38γ isoform has a distinct functional role in skeletal muscle adaptation through its regulatory function on PGC-1α, particularly the *Pgc-1*α gene transcription, we employed electric pulse-mediated gene transfer to transiently transfect adult skeletal muscle with empty control vector (pCI-neo) or plasmid DNA containing epitope-tagged (FLAG) dominant negative forms of p38 isoforms in mouse tibialis anterior muscles (TA) followed by motor nerve stimulation. The motor nerve stimulation led to muscle contraction that mimics an acute bout of endurance exercise. This gene transfer method resulted in at least 60% of the myofibers expressing the transgene as shown by transfection with a plasmid DNA encoding enhanced green fluorescent protein (pEGFP) (Fig. 3A). Although the epitope-tagged dominant negative forms of p38 proteins can be easily detected by immunoblot analysis in the TA muscle (Fig. 3B), but only that of p38γ blocked motor nerve stimulation-induced *Pgc-1α* mRNA expression (Fig. 3C). TA muscles transfected with empty vector, dominant negative forms of p38α and p38β had 3.3-, 3.8- and 3.6-fold (p<0.001, 0.01 and 0.001, respectively) increases in *Pgc-1α* mRNA following motor nerve stimulation; however, TA muscles transfected with the dominant negative p38γ showed significantly attenuated expression (1.4-fold, p = 0.35) (Fig. 3C). Thus, dominant negative p38γ, but not those of p38α and p38β, is capable of blocking endurance exercise-induced transcriptional activation of the *Pgc-1α* gene in skeletal muscle, providing additional mechanistic evidence supporting an obligatory role of p38γ in skeletal muscle metabolic adaptation.

### Muscle-Specific Deletion of the p38γ Gene Leads to Altered Target Gene Expression in Response to Motor Nerve Stimulation

**Figure 4:**
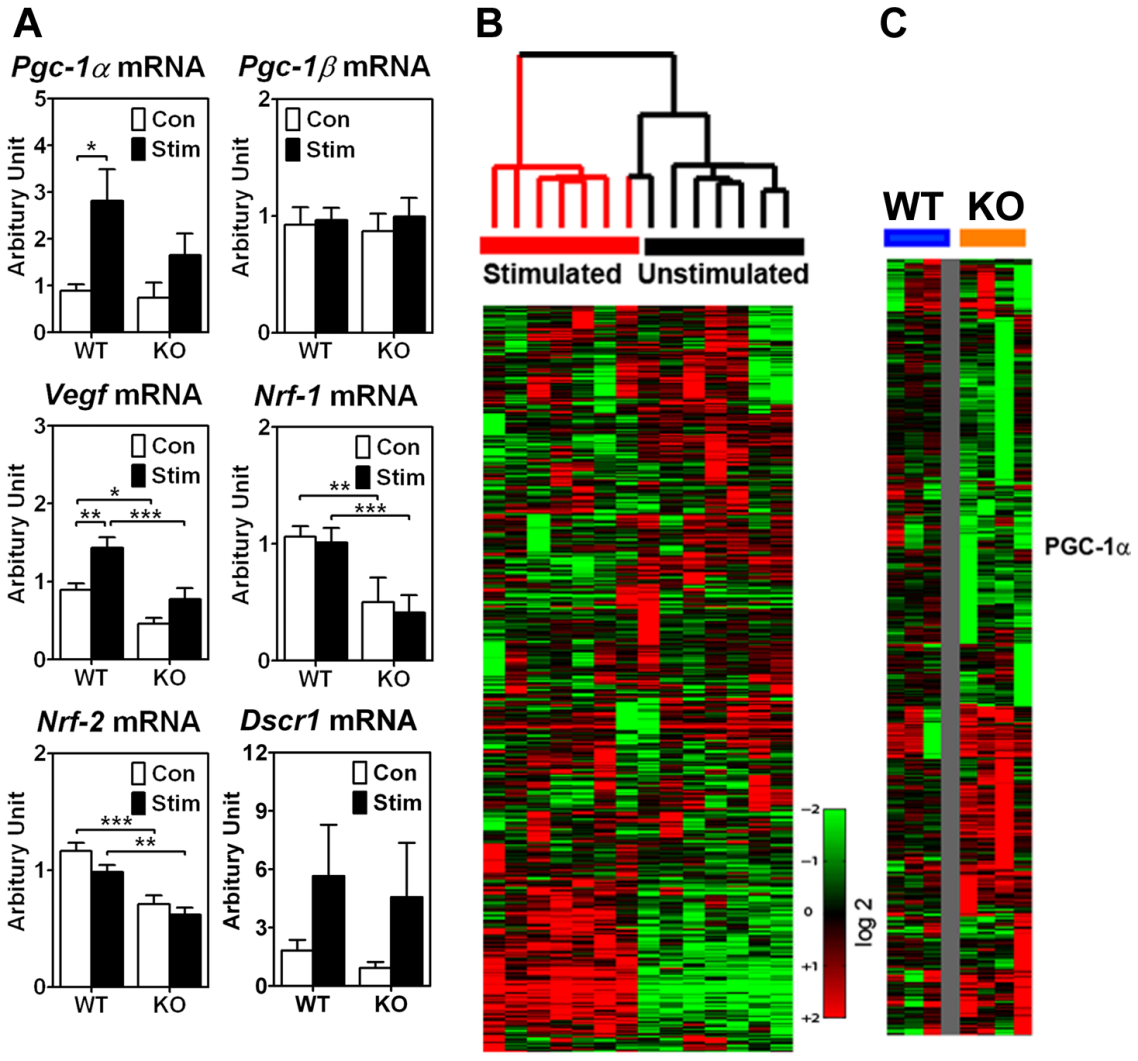
Muscle-specific deletion of the p38γ gene results in alterations in endurance exercise-induced gene expression. Wild type and p38γ KO mice were subjected to motor nerve stimulation (10 Hz, 2 hours) via the deep peroneal nerve, which innervates the tibialis anterior muscle. One hour following motor nerve stimulation, both the stimulated tibialis anterior and the contralateral control tibialis anterior muscles were harvested for total RNA isolation and analyzed by real-time PCR and Affymetrix microarray analyses. A) Real-time PCR analysis for *Pgc-1α, Pgc-1β, Vegf, Nrf-1*, *Nrf-2* and *Dscr1* mRNA (n = 5–6). *, ** and *** denote p<0.05, 0.01 and 0.001, respectively; B) Hierarchical analysis for the 932 genes with expression variations of at least 2 fold in one sample, showing the effects of motor nerve stimulation; and C) Motor-nerve stimulation-induced changes in gene expression in wild type and p38 KO mice are compared after 601 genes were selected based on expression variations of at least 2 fold in one sample. The *Pgc-1α* gene is among the genes with reduced induction in response to motor nerve stimulation in MKO mice.

Orchestrated signaling-transcription events, which could be elicited by as little as a single bout of endurance exercise, play important roles in skeletal muscle adaptation [21]. To begin to identify the downstream target genes of the p38γ MAPK pathway related to endurance exercise training, we performed real-time PCR analysis for TA muscles that were stimulated *via* the deep peroneal nerve (10 Hz for 2 hours) followed by 1 hour of resting period and compared with the contralateral control TA muscle. Motor nerve stimulation-induced *Pgc-1α* and *Vegf* mRNA expression in TA muscle was significantly attenuated in p38γ MKO mice (Fig. 4A), whereas neither the motor nerve stimulation nor the p38γ gene deletion in skeletal muscle had significant impact on *Pgc-1β* mRNA expression. Muscle-specific deletion of the *p38γ* gene also led to reduced nuclear respiratory factor-1 (*Nrf-1)* and *Nrf-2* mRNA expression, but their expression was influenced by motor nerve stimulation. On the contrary, motor-nerve stimulation-induced Down syndrome critical region 1 (*Dscr1)* mRNA expression did not appear to be affected by the deletion of the p38γ gene in skeletal muscle (Fig. 4A), suggesting activation of the calcineurin pathway, which has been implicated in fiber-type specification [22], [23], [24], is not dependent on the p38*γ* MAPK. This observation further supports the notion that endurance exercise-induced activation of p38*γ* MAPK is a separate regulatory event from contractile adaptation in skeletal muscle.

We then performed microarray analysis using the Affymetrix Mouse Genome 430A 2.0 Array in p38*γ* MKO muscles to evaluate global gene expression in response to increased contractile activities. The array data were first normalized by robust multichip average (RMA). The data discussed in this publication have been deposited in NCBI's Gene Expression Omnibus [25] and are accessible through GEO Series accession number GSE17620 (http://www.ncbi.nlm.nih.gov/geo/query/acc.cgi?acc=GSE17620). Motor nerve stimulation clearly induced dramatic changes in gene expression leading to consistent grouping of the contralateral control muscles away from stimulated muscles using unsupervised clustering analysis (Fig. 4B). One cluster of 241 probe sets was consistently elevated in response to motor nerve stimulation (Fig. 4B and Table S1). When these genes were analyzed for the enrichment of biological processes, they were found to be enriched for genes functioning in the MAPK signaling, transforming growth factor-β (TGF-β) signaling, focal adhesion and extracellular matrix (ECM)-receptor interaction (Table S2), all of which have previously shown to be readily up-regulated by increased contractile activities [21], [26], [27]. The findings of enrichment of enhanced gene expression functioning in the MAPK signaling again support its functional importance in skeletal muscle remodeling.

To identify the differences in the motor nerve stimulation-induced gene expression, we performed zero transformation between the samples from the wild type and p38γ MKO mice and identified 601 genes that are differentially regulated at least 2 fold in two samples between wild type and p38γ MKO mice, which are presented as supplemental data (Table S3 and Table S4) in *PLoS One* on-line. *Pgc-1α* (detected by 3 different probes) along with many other genes, such as nicotinic cholinergic receptor gamma peptide (*Chrng*), fibroblast growth factor 4 (*Fgf4*), embryonic skeletal muscle myosin heavy polypeptide 3 (*Myh3*), myosin light polypeptide 3 (*Myl3*), Ca2+/calmodulin-dependent protein kinase kinase 2 (*Camkk2*), myogenin (*Myog*), peroxisome proliferator activated receptor α (*Ppara*), acyl-CoA synthetase long-chain family 1 (*Acsl1*) and 3-hydroxybutyrate dehydrogenase (*Bdh1*), showed attenuated induction by motor nerve stimulation in p38γ MKO mice (Fig. 4C). These genes are possible target genes of the p38γ MAPK pathway in skeletal muscle.

In the same analysis, many genes, including SRY-box containing gene 4 (*Sox4*), *Sox9*, mitogen-activated protein kinase kinase kinase kinase 4 (*Map4k4*), β-myosin heavy chain (*Myh7*), myosin light chain 2 (*Myl2*), cardiac troponin C (*Tnnc1*), NADPH oxidase 4 (*Nox4*), homer homolog 1 (*Homer1*), suppressor of cytokine signaling 3 (*Socs3*), Jun-B oncogene (*Junb*), heat shock protein 1A (*hspa1a*), heat shock protein 1B (*Hspa1b*), ankyrin repeat domain (Ankrd1), tumor necrosis factor receptor superfamily member 12a (*Tnfrsf12a*), FBJ osteosarcoma oncogene (*Fos*), activating transcription factor 3 (*Atf3*), CCAAT/enhancer binding protein (C/EBP) δ (*Cebpd*) and growth arrest and DNA-damage-inducible 45 β and γ (*Gadd45b* and *Gadd45g*), showed significantly enhanced expression over the wild type background following motor nerve stimulation. These are genes that are probably repressed by the p38γ MAPK pathway or become more sensitive to motor nerve stimulation in the absence of p38γ function in skeletal muscle.

Many genes with attenuated induction in p38γ MKO were significantly enriched in the Gene Ontology (GO) of immune and inflammatory responses, chemotaxis, catabolism and other cellular processes (Table S5). On the other hand, genes with exaggerated induction were enriched in GO of organogenesis, development, morphogenesis as well as activation of MAPKKK (Table S6). These analyses provide functional perspective of the p38γ MAPK pathway in skeletal muscle.

A statistically significant number of genes that contain the binding sites of modulator recognition factor 2 (MRF2), c-Myc:Max complex (MYC:MAX), E2F, complex of Lmo2 (LMO2COM) , nuclear respiratory factor 2 (NRF-2), cAMP response element binding (CREB), activating transcription factor (ATF), serum response factor (SRF), MYOD, GATA1 and VMYB transcription factors were enriched with attenuated up-regulation in p38γ MKO mice in response to motor nerve stimulation (Table S7). Target genes bearing these binding sites are known to be important for various cellular processes, such as stress response, cell growth and development, and mitochondrial gene expression. All these findings collectively provide evidence that activation of p38γ plays a functional role in endurance exercise-induced genetic reprogramming in skeletal muscle. Considering these gene expression findings and functional findings of attenuated mitochondrial biogenesis and angiogenesis, we conclude that p38γ functions in endurance exercise training-induced metabolic adaptation. The global gene expression analysis in skeletal muscle with muscle-specific deletion of the p38γ gene following motor nerve stimulation made it possible to identify contractile activity-dependent p38γ target genes.

## Discussion

It has been known since antiquity that regular endurance exercise improves physical performance and brings about health benefits. Recent advancement in molecular genetics, such as the technologies for transgenic and knockout mice, has allowed us to gain significantly improved understanding of the underlying molecular and signaling mechanisms in skeletal muscle plasticity with a great appreciation of the importance of an orchestrated signaling-gene regulation network [8], [20], [22], [23], [28], [29], [30], [31], [32], [33], [34], [35], [36], [37]. In this study, we have employed skeletal muscle-specific gene disruption approach in animal models and ascertained the functional role and isoform-specificity of the p38 MAPK pathway in endurance exercise-induced skeletal muscle adaptation. Biochemical and gene expression evidence supports that p38γ MAPK is required for endurance exercise-induced mitochondrial biogenesis and angiogenesis, but not fiber type transformation. These findings provide evidence that skeletal muscle metabolic adaptations could be genetically separated from the contractile adaptation.

The importance of the p38 MAPK pathway in exercise-induced skeletal muscle adaptation has been implicated in numerous previous studies [1], [2], [3], [4], [5], [8], [38], [39]. Particularly, it has been shown that various types of contractile activities activate the p38 MAPK pathway in skeletal muscles [2], [4], [38], and genetic activation of the p38 MAPK pathway promotes the *Pgc-1α* gene expression and mitochondrial biogenesis in skeletal muscle [8]. These studies suggest a possible link between the activity of the p38 MAPK and PGC-1*α* expression and function in experimental settings, but have not determined the functional role of the endogenous p38 MAPK where a specific isoform of the p38 genes is ablated specifically in skeletal muscle. We obtained mice with deletions of each of the p38 genes in skeletal muscle (p38 MKO) according to an established approach [13], [40]. Surprisingly, deletion of the p38 genes had no discernable effects on endurance exercise-induced IIb-to-IIa fiber type transformation in plantaris muscles. Disruption of the p38γ gene in skeletal muscle did not affect motor nerve stimulation-induced *Dscr1* mRNA expression, which is indicative of activation of the Ca^2+^-dependent calcineurin pathway that is essential for fiber-type specification [22], [23], [24]. These findings suggest that activation of the p38 MAPK pathway in skeletal muscle is not required for endurance exercise training-induced IIb-to-IIa fiber type transformation (contractile adaptation).

Endurance exercise-induced activation of the p38 MAPK pathway and the consequent activation of PGC-1*α* at the transcriptional and post-transcriptional levels are considered critical for skeletal muscle adaptation. The findings of fiber type transformation in p38 MKO mice are quite surprising as overexpression of the *Pgc-1α* gene in skeletal muscle causes significant glycolytic-to-oxidative fiber-type transformation [33]. Leick *et al.* has recently reported in a whole body knockout mouse model that PGC-1α is not mandatory for exercise- and training-induced adaptive gene responses in skeletal muscle. Our finding that all of the muscle-specific p38 MKO mouse lines had normal IIb-to-IIa fiber-type transformation may also suggest that PGC-1α function is at least not required for contractile adaptation. Alternatively, other regulatory factor(s), such as PGC-1β, may play a redundant role as a compensatory safeguard. PGC-1β has been shown to be important for formation of type IIx fibers [41].

The functional importance of p38γ MAPK-PGC-1α regulatory axis in metabolic adaptations was obtained in this study following long-term voluntary running. Muscle-specific disruption of the p38γ gene, but not that of the p38α or p38β gene, significantly attenuated endurance exercise-induced COX IV and Cyt c expression in p38γ MKO mice, providing genetic evidence that the p38γ gene is required for endurance exercise-induced mitochondrial biogenesis in skeletal muscle. The attenuated *Pgc-1α* gene transcription in response to increased contractile activity in skeletal muscle of p38γ MKO mice and in skeletal muscle transfected with dominant negative p38γ, but not that of dominant negative p38α and p38β, further supports this notion. These biochemical and gene expression findings are consistent with the notion that p38γ MAPK are upstream of the *Pgc-1α* transcription in the cascade of signaling transcription coupling between neuromuscular activity and mitochondrial biogenesis.

PGC-1α plays a role in hypoxia-induced angiogenesis in skeletal muscle through its regulatory function on the *Vegf* gene expression [20]. Our findings that endurance exercise-induced angiogenesis in skeletal muscle is attenuated in p38γ MKO mice along with impaired *Pgc-1α* and *Vegf* transcriptional activation support the importance of p38γ-PGC-1α regulatory axis in the adaptation of the vascular system in skeletal muscle. Consistent with this notion was the finding that global gene disruption of the *Pgc-1*α gene resulted in impaired VEGF expression following endurance exercise training [42]. These findings together with the findings in this study that none of the genetic manipulations affected endurance exercise-induced fiber-type transformation genetically segregate the metabolic adaptations from contractile adaptation in genetic models with muscle-specific gene deletion in mice.

PGC-1α has an important auto-regulatory function in skeletal muscle [43], i.e. PGC-1α positively regulate *Pgc-1α* gene transcription. Since endurance exercise acutely stimulates *Pgc-1α* transcription, which could be mediated by a signaling–transcription cascade(s) [8] and/or an activation of PGC-1α [6], *Pgc-1α* mRNA expression could serve as a surrogate for these regulatory events. Both real-time PCR and microarray analyses in this study have shown that induced *Pgc-1α* mRNA by motor nerve stimulation was significantly diminished in p38γ MKO mice, supporting that p38γ MAPK is required for *Pgc-1α* transcription and/or PGC-1α activity. Our finding that muscle-specific overexpression of a dominant negative form of p38γ, but not that of p38α and p38β, blocks motor nerve stimulation-induced *Pgc-1α* mRNA provided independent verification of these findings.

Previous studies have shown that a single bout of endurance exercise in mice is sufficient to induces robust changes in global gene expression, suggesting the importance of a signaling-transcription network in the genetic reprogramming [21]. A unique feature of our studies is the comparison of stimulation-induced global gene expression between p38γ MKO and the wild type littermates. We found a subset of genes with diminished or loss of induction upon motor nerve stimulation in p38γ MKO mice. Our data confirmed once again that the *Pgc-1α* gene (3 independent probes) is a target of the p38γ MAPK pathway in skeletal muscle in a loss-of-function genetic model, which is not only consistent with but also provides additional insights into the previous findings [8], [11]. In the same analysis, genes that encode proteins in contractile apparatus (*Myh3* and *Myl3*), signaling and transcriptional regulation (*Hif3*, *Camkk2*, *Myog* and *Ppara*), metabolism (*Bdh1* and *Acsl1*), neuromuscular junction (*Chrng*) and humoral regulation (*Fgf4* and *Il1b*) along with many other genes are now confirmed to be controlled by the p38 MAPK pathway in skeletal muscle.

Additional bioinformatics analysis showed enrichment of p38γ MAPK-dependent genes with certain transcription factor binding sites, among which CREBATF and CREB have been shown to control *Pgc-1α* transcription [8], [11], [43], [44], and NRF-2 has been suggested to function with PGC-1 family co-activators in the coordinate regulation of nucleus-encoded mitochondrial transcription factors [45]. These findings suggest the importance of p38γ MAPK in metabolic adaptation through these transcription factors and their target genes. Several other transcription factors, including MRF2, MYC:MAX, E2F, LMO2COM, SRF, MYOD, GATA1 and VMYB, are implicated to have similar functions, which requires further investigation.

**Figure 5:**
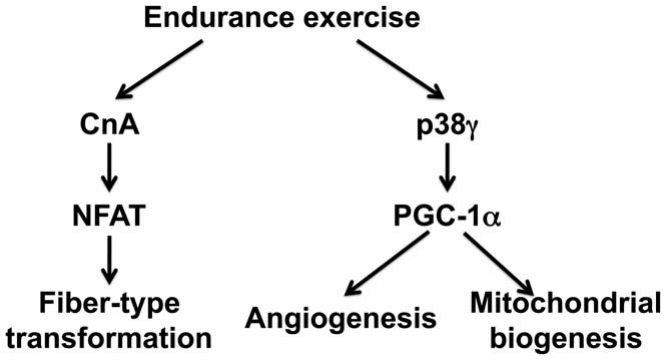
Signaling and transcriptional control of endurance exercise-induced contractile adaptation and metabolic adaptation. This is a schematic presentation of a hypothetical model for endurance exercise-induced signaling/transcriptional control of skeletal muscle adaptation. Endurance exercise on one hand induces activation of the Ca^2+^-dependent calcineurin (CnA)-NFAT pathway in control of fiber-type transformation and on the other hand activates p38γ, which promotes PGC-1α activity and expression in control of mitochondrial biogenesis and angiogenesis.

In summary, we have obtained direct evidence for the functional importance of p38γ in endurance exercise-induced activation of the *Pgc-1α* gene in skeletal muscle. Our observations together with previous studies [8], [11], [12] suggest that calcineurin (CnA)-nuclear factor of activated T-cells (NFAT) regulatory axis controls fiber-type transformation whereas p38γ-PGC-1α regulatory axis controls mitochondrial biogenesis and angiogenesis in endurance exercise-induced skeletal muscle adaptation (Fig. 5). The findings from this study specifically raised the possibility of using a specific p38γ activator to promote skeletal muscle metabolic function.

## Materials and Methods

### Ethics Statement

All animal protocols were approved by the Duke University Institutional Animal Care and Use Committee.

### Animals

Adult male C57BL/6J mice (8-weeks old) were supplied commercially (Jackson Laboratory) and housed in a temperature-controlled (21°C) facility with a 12-h light/12-h dark cycle. Mice were supplied with normal chow (Purina Chow) and water *ad libitum*. Mice with muscle-specific knockout of the p38 MAPK isoform genes were generated by crossbreeding myogenin-*Cre* mice (generous gift from Dr. Eric Olson) with floxed-p38α, p38β, or p38γ mice (kindly provided by Boehringer Ingelheim Pharmaceuticals, Inc.).

### Genotyping

Genomic DNA was isolated by incubating tail tissue overnight at 55°C in proteinase K buffer (1204241, InvivoGen). The genomic DNA was suspended in tris-EDTA buffer and 2 µl of the DNA was used for PCR to determine the genotypes. The following PCR primers were used. *Cre* transgene: 5′-AGGTTCGTTCACTCATGGA-3′ and 5′-TCGACCAGTTTAGTTACCC-3′; loxP-flanked *Pgc1α* allele: 5′-TCCAGTAGGCAGAGATTTATGAC-3′ and 5′-TGTCTGGTTTGACAATCTGCTAGGTC-3′ loxP-flanked p38α allele: 5′-TCCTACGAGCGTCGGCAAGGTG-3′ and 5′-ACTCCCCGAGAGTTCCTGCCTC-′3; loxP-flanked p38β allele: 5′-TCGCTCCAGCTGCTTCTGTGGA-′3, 5′-AACCCGGATGGCTGACTGTTCC-′3 and 5′-CTGAGGCGGAAAGAACCAGCTG-′3; loxP-flanked p38γ allele: 5′-CCAGGAGGTGACCAAAACGGC-′3, 5′-TGGGCTGCGAAGGTAGAGGTG-′3 and 5′-CTGAGGCGGAAAGAACCAGCTG-′3. Sequential denaturing (96°C for 30 sec), annealing (55°C for 30 sec) and extension (72°C for 30 sec) were repeated 30 times for genotyping the *Cre* transgene. To genotype for the alleles of the p38 genes, the following parameters were used: 30 cycles of denaturing (94°C for 30 sec), annealing (68°C for 2 min) and extension (72°C for 45 sec).

### Voluntary Running

Mice were subjected to voluntary running (4 weeks) in cages equipped with a running wheel of 0.357 m circumference [19], [46]. Running data was recorded and quantified using Dataquest ART Gold Acquisition Software 2.2. Running wheels were locked after the last episode of running for a 48-hour period prior to sacrifice and muscle harvest.

### Motor Nerve Stimulation

The experimental procedure was performed under anesthesia as described previously [47]. Motor nerve stimulation started within 30 minutes after the surgery and lasted for 2 hours at 10 Hz (0.25 ms duration). The amplitude of the electric pulses was adjusted between 1–3 V to achieve consistent contractions without damage to the nerve. Mice were allowed to recover for 1 hour prior to the harvest of the tibialis anterior muscles.

### Gene Transfer

Electric pulse-mediated gene transfer was performed as described previously except that each of the tibialis anterior muscles received 100 µg (2 µg/µl in normal saline) plasmid DNA [11].

### Real-Time PCR

Total RNA was extracted from tibialis anterior muscle using TRIzol (Invitrogen) according to the manufacturer's instructions. cDNA was generated by a reverse transcription reaction, and real-time PCR was then performed using an ABI Prism 7000 sequence detection system (Applied Biosystems) with an initial holding temperature at 50°C for 2 min, 95°C for 10 min, followed by 40 cycles of 95°C for 15 sec and 60°C for 1 min. The threshold cycle (Ct) was determined using the supplied software, and standard curves were established to quantify the products. The data was normalized to *18s* rRNA.

### Microarray Analysis

Biotinylated cRNA were prepared according to the standard Affymetrix protocol from 2 µg total RNA (Expression Analysis Technical Manual, 2001, Affymetrix). Following fragmentation, 10 µg of cRNA were hybridized for 16 hours at 45°C on GeneChip Mouse430A 2.0. GeneChips were washed and stained in the Affymetrix Fluidics Station 450. Affymetrix GeneChips were scanned using the Affymetrix Scanner 3000 7G. The data were analyzed with Affymetrix GeneChip Command Console Software (AGCC) using Affymetrix default analysis settings and global scaling as normalization method. The trimmed mean target intensity of each array was arbitrarily set to 500. All data is MIAME compliant, and the raw data has been deposited in a MIAME compliant database, the Gene Expression Omnibus (GEO) database, as detailed on the MGED Society website http://www.mged.org/Workgroups/MIAME/miame.html and is available at http://www.ncbi.nlm.nih.gov/geo/query/acc.cgi?acc=GSE17620.

### Immunoblot Analysis

Skeletal muscle samples were subjected to immunoblot analysis as described previously [47]. The following antibodies were used: COX IV (ab14744, Abcam), Cyt c (4272, Cell Signaling Technology), p38α (MAB869, R&D Systems), p38γ (MAB1347, R&D Systems), P-p38 (9216, Cell Signaling Technology), Gapdh (ab9484, Abcam), α-tubulin (ab11304, Abcam). Proteins were quantified by using Scion Image software.

### Fiber-Typing

Fiber-type analysis was determined by immunohistochemistry techniques as described previously [19]. Each type of the myofibers were counted for the entire plantaris muscle cross section, and presented as percentage of the total fibers.

### Determination of Capillary Density

Similar immunofluorescence procedures as described above were followed by using rat anti-CD31 (MCA1364; Serotec, Raleigh, NC). Total number of capillaries and the surface area of the entire cross-section for each muscle were measured with Scion Image software (Scion, Frederick, MD) and presented as capillary density (capillaries/mm^2^).

### Statistical Analysis

Data are expressed as mean ± S.E. Statistical significance (p<0.05) was determined by two-way repeated measures ANOVA for nerve-stimulation experiment and by two-way ANOVA for running experiment followed by Newman-Keuls test.

## Supporting Information

Figure S1 Voluntary running in muscle-specific p38α, p38β, and p38γ MKO mice. A) Adult mice (8 weeks of age) were subjected to voluntary running for 4 weeks. Daily running distance was calculated (n = 5–8); and B) Heart weight (normalized by body weight) in sedentary (Sed) and exercise-trained (Ex) wild type (WT), p38α, p38β, and p38γ MKO mice (n = 5–8).
(7.54 MB TIF)

Figure S2 Muscle-specific deletion of the p38α, p38β, or p38γ gene does not affect exercise-induced fiber-type transformation. Mice with skeletal muscle-specific deletion of the p38 genes were obtained by crossbreeding between myogenin-Cre TG mice and genetically modified mice with the p38 alleles flanked by loxP sites. Wild type, p38α, p38β, and p38γ MKO mice were subjected to 4 weeks of voluntary running (Ex) with sedentary mice (Sed) as control followed by immunoblot analysis in plantaris muscles for quantification of myosin heavy chain IIa (MHC IIa) protein (n = 5–8). *, ** and *** denote p<0.05, 0.01 and 0.001, respectively.
(2.84 MB TIF)

Table S1 The probesets, gene names and symbols for the cluster of genes whose expression is up-regulated in skeletal muscle in response to motor nerve stimulation in tibialis anterior muscle.
(0.05 MB XLS)

Table S2 The enriched KEGG pathways in the gene cluster in skeletal muscle induced by motor nerve stimulation.
(0.01 MB XLS)

Table S3 The probesets, gene names and symbols for the cluster of genes whose stimulation-induced change is reduced in the p38γ MKO mice when compared with wild type littermates.
(0.06 MB XLS)

Table S4 The probesets, gene names and symbols for the cluster of genes whose stimulation-induced change is increased in the p38γ MKO mice when compared with wild type littermates.
(0.05 MB XLS)

Table S5 The GO terms significantly enriched for genes whose stimulation-induced change is reduced in the p38γ MKO mice when compared with wild type littermates.
(0.03 MB XLS)

Table S6 The GO terms significantly enriched for genes whose stimulation-induced change is increased in the p38γ MKO mice when compared with wild type littermates.
(0.02 MB XLS)

Table S7 The binding sites for transcriptional factors significantly enriched for genes whose stimulation-induced change is reduced in the p38γ MKO mice when compared with wild type littermates.
(0.03 MB XLS)
