# Supplementary figures and images for: p38γ Mitogen-Activated Protein Kinase Is a Key Regulator in Skeletal Muscle Metabolic Adaptation in Mice
Source: PLoS One. 2009 Nov 20;4(11):e7934. doi: 10.1371/journal.pone.0007934 (PMC2775956; doi:10.1371/journal.pone.0007934)

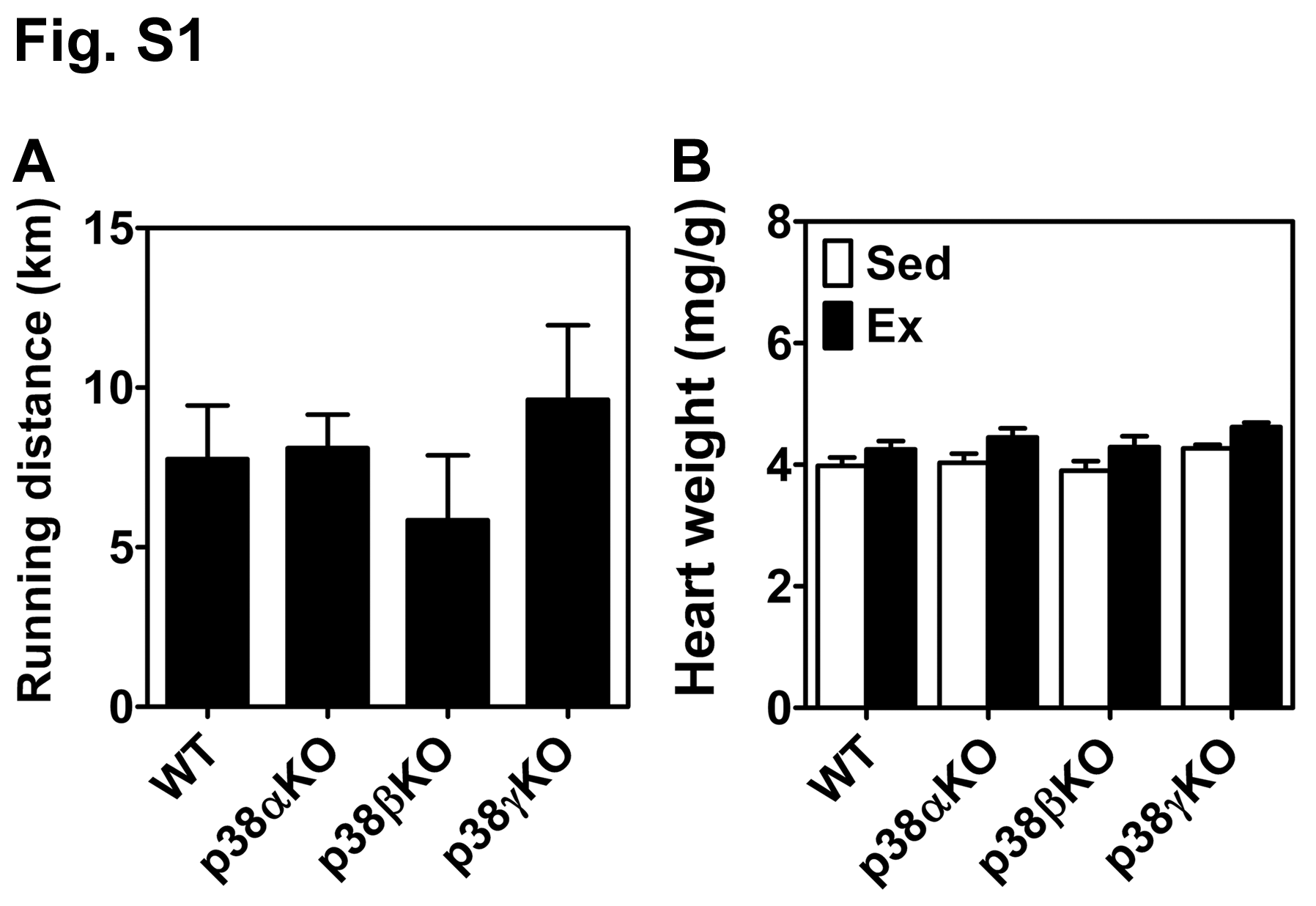

Supplement: Figure S1 — Voluntary running in muscle-specific p38α, p38β, and p38γ MKO mice. A) Adult mice (8 weeks of age) were subjected to voluntary running for 4 weeks. Daily running distance was calculated (n = 5–8); and B) Heart weight (normalized by body weight) in sedentary (Sed) and exercise-trained (Ex) wild type (WT), p38α, p38β, and p38γ MKO mice (n = 5–8). (7.54 MB TIF) [file pone.0007934.s001.tif]

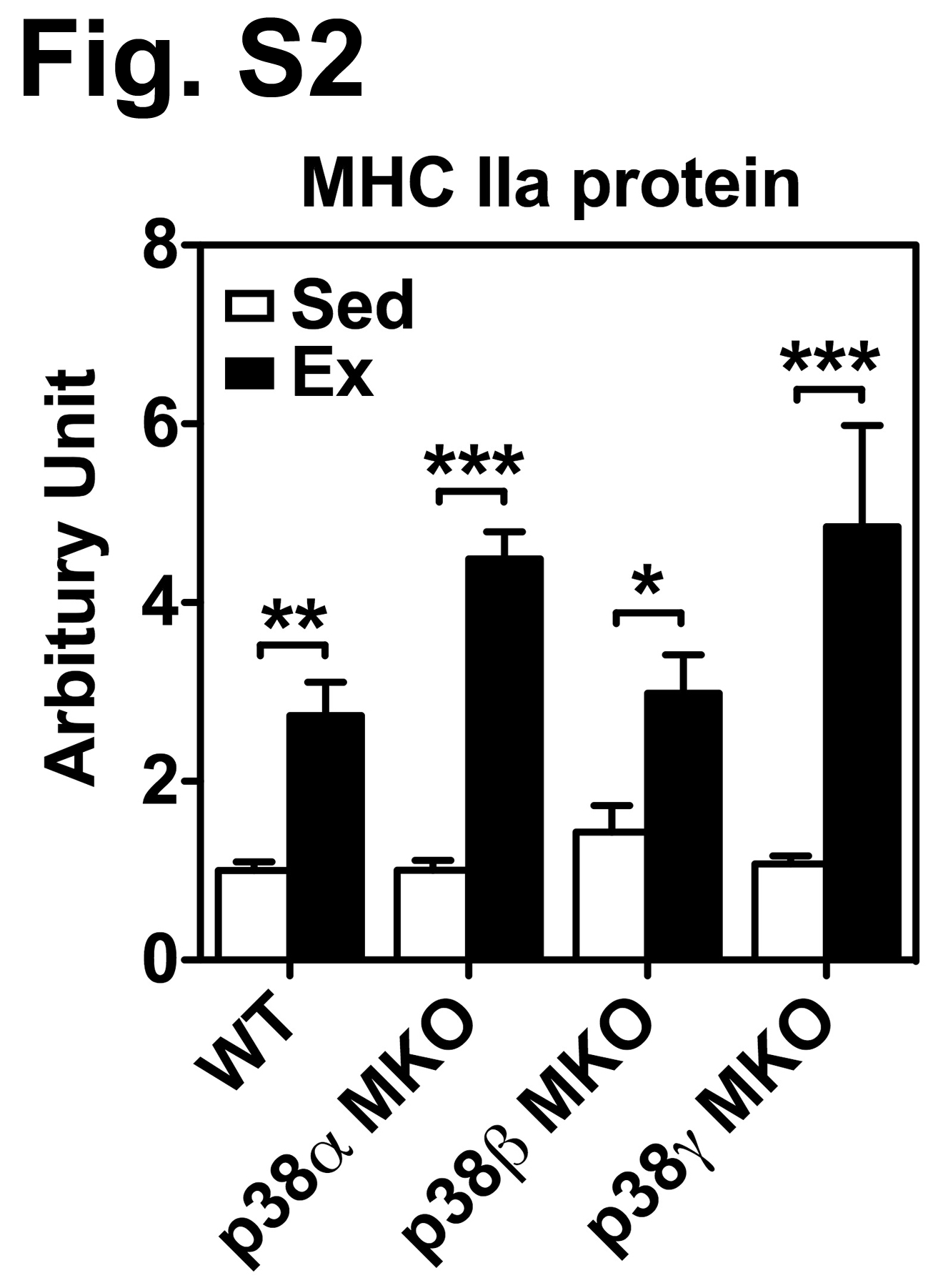

Supplement: Figure S2 — Muscle-specific deletion of the p38α, p38β, or p38γ gene does not affect exercise-induced fiber-type transformation. Mice with skeletal muscle-specific deletion of the p38 genes were obtained by crossbreeding between myogenin-Cre TG mice and genetically modified mice with the p38 alleles flanked by loxP sites. Wild type, p38α, p38β, and p38γ MKO mice were subjected to 4 weeks of voluntary running (Ex) with sedentary mice (Sed) as control followed by immunoblot analysis in plantaris muscles for quantification of myosin heavy chain IIa (MHC IIa) protein (n = 5–8). *, ** and *** denote p<0.05, 0.01 and 0.001, respectively. (2.84 MB TIF) [file pone.0007934.s002.tif]
